# Transcriptome analysis showed that tomato-rootstock enhanced salt tolerance of grafted seedlings was accompanied by multiple metabolic processes and gene differences

**DOI:** 10.3389/fpls.2023.1167145

**Published:** 2023-06-02

**Authors:** Xiaolei Wu, Ding Yuan, Xinyu Bian, Ruixiao Huo, Guiyun Lü, Binbin Gong, Jingrui Li, Sichao Liu, Hongbo Gao

**Affiliations:** ^1^ College of Horticulture, Hebei Agricultural University, Key Laboratory of North China Water-saving Agriculture, Ministry of Agriculture and Rural Affairs, Hebei Key Laboratory of Vegetable Germplasm Innovation and Utilization, Collaborative Innovation Center of Vegetable Industry in Hebei, Baoding, China; ^2^ Chengde Vegetable Technology Promotion Station, Chengde, China

**Keywords:** transcriptome, NaCl stress, grafting, Na + transportation, amino acid accumulation, plant hormone signal transduction

## Abstract

**Introduction:**

Grafting is a commonly used cultural practice to counteract salt stress and is especially important for vegetable production. However, it is not clear which metabolic processes and genes are involved in the response of tomato rootstocks to salt stress.

**Methods:**

To elucidate the regulatory mechanism through which grafting enhances salt tolerance, we first evaluated the salt damage index, electrolyte permeability and Na^+^ accumulation in tomato (*Solanum lycopersicum* L.) leaves of grafted seedlings (GSs) and nongrafted seedlings (NGSs) subjected to 175 mmol·L^− 1^ NaCl for 0-96 h, covering the front, middle and rear ranges.

**Results:**

Compared with the NGS, the GSs were more salt tolerant, and the Na^+^ content in the leaves decreased significantly. Through transcriptome sequencing data analysis of 36 samples, we found that GSs exhibited more stable gene expression patterns, with a lower number of DEGs. *WRKY* and *PosF21* transcription factors were significantly upregulated in the GSs compared to the NGSs. Moreover, the GSs presented more amino acids, a higher photosynthetic index and a higher content of growth-promoting hormones. The main differences between GSs and NGSs were in the expression levels of genes involved in the BR signaling pathway, with significant upregulation of *XTHs*. The above results show that the metabolic pathways of “photosynthetic antenna protein”, “amino acid biosynthesis” and “plant hormone signal transduction” participate in the salt tolerance response of grafted seedlings at different stages of salt stress, maintaining the stability of the photosynthetic system and increasing the contents of amino acids and growth-promoting hormones (especially BRs). In this process, the transcription factors *WRKYs, PosF21* and *XTHs* might play an important role at the molecular level.

**Discussion:**

The results of this study demonstrates that grafting on salt tolerant rootstocks can bring different metabolic processes and transcription levels changes to scion leaves, thereby the scion leaves show stronger salt tolerance. This information provides new insight into the mechanism underlying tolerance to salt stress regulation and provides useful molecular biological basis for improving plant salt resistance.

## Introduction

Soil salinization is one of the main abiotic stresses affecting plant growth and development worldwide, with an estimated 833 million hectares of land currently affected by salinity, accounting for 8.7% of the Earth’s area and 20% of irrigated land (Said et al., 2022; Zhang et al., 2022). In recent years, the protected vegetable industry has developed vigorously, but current environmental conditions and overapplication of chemical fertilizers have caused secondary soil salinization to increase daily. Investigations have shown that the total salt content of protected vegetable fields is on average 69.3% higher than that of open-field vegetable fields in China (Huang et al., 2016). Therefore, it is particularly important for protected crops to explore ways to alleviate salt stress.

Na^+^ and Cl^−^ are not essential mineral but are the main ions causing salt stress injury to plants (Ramesh et al., 2015). Soil salinity can hinder plant productivity and inhibit physiological and biochemical processes (Marschner, 1995; Shi et al., 2022), including photosynthesis and membrane integrity. Soil salinity can also lead to chlorophyll degradation, membrane lipid peroxidation, and ion balance disruption, thereby reducing membrane fluidity and selectivity (Bulgari et al., 2019; Hashem et al., 2019; Essalimi et al., 2022; Desoky et al., 2019; Sitohy et al., 2020). Therefore, improving the salt tolerance of plants has become a popular research topic in recent years. This research mainly focuses on the selection and breeding of salt-tolerant varieties, the application of exogenous substances and the use of reasonable irrigation systems. Among these methods, the screening and utilization of salt-tolerant varieties is the most effective way to improve the salt tolerance of greenhouse-grown crops. Compared with cultivated varieties, rootstock varieties are usually more salt tolerant (Penella et al., 2017), but there are few studies on the mechanism underlying the salt tolerance of and identifying salt-tolerant rootstock varieties.

Grafting, which was first applied in melon production, is a commonly used cultural practice to counteract salt stress in practical production (Tian et al., 2012). Cucumber and watermelon were used as experimental materials to improve the salt tolerance of vegetables by grafting. Grafting can enhance the salt tolerance of plants at multiple levels, including regulating the absorption and distribution of K^+^ and Na^+^ (Estan et al., 2005). After grafting, more Na^+^ is stored in the root system, and the selective absorption capacity of K^+^ strengthens (Goreta et al., 2008). Tomato (*Solanum lycopersicum* L.) is one of the most widely cultivated, moderately salt-sensitive horticultural crop species worldwide, and it can be used for the development of the vegetable industry in saline-alkali areas after variety improvement or cultivation technology innovation. Therefore, it is of great significance to explore the salt-alkali tolerance mechanism of tomato and improve the salt-alkali tolerance of tomato for the development of the vegetable industry in saline-alkali areas. Compared with that of melon rootstocks, the screening and utilization of tomato rootstocks and the production and application of grafted seedlings (GSs) occurred later, and the improvement of the salt tolerance of tomato seedlings by grafting is manifested through many physiological factors. However, there have been few reports on the research and utilization of tomato rootstock salt tolerance mechanisms in grafted tomato seedlings. Therefore, it is important to determine which metabolic processes and genes are involved in the response of tomato rootstocks to salt stress to analyze the mechanism of stress resistance of grafting.

The development of molecular biological technology, particularly transcriptome sequencing (RNA-seq) technology, makes it possible to understand the salt resistance mechanism of tomato and provides a technical means for understanding salt tolerance mechanisms at the molecular level. At present, a large number of genes induced in response to salt stress have been isolated and identified (Seo et al., 2012; Safdar et al., 2013), mainly sodium ion transport-related genes, including members of the NHX (Na^+^/H^+^ antiporter) family, and osmotic stress resistance-related genes, including the zinc finger protein-coding gene and the trehalose-6-phosphate synthase gene (Li et al., 2013; Sun et al., 2014). It is reported that multiple hormone regulatory networks during grafting involve auxin, cytokinin, abscisic acid, and gibberellic acid pathways, as well as a variety of transcription factors, which are involved in complex growth induction processes. However, there are few studies on how various hormones change and gene changes during signal transduction under salt stress(Chen et al., 2020). Moreover, it is not clear which metabolic pathway genes are differentially expressed to elicit the differences in leaf salt stress damage between GSs of salt-tolerant rootstocks and NGS.

To explore the molecular and physiological mechanism of salt tolerance induced by grafting tomato with salt-tolerant rootstock under salt stress, we investigated differences in the salt stress response and Na^+^ content of GSs and NGSs. Furthermore, the transcriptome profiles of both of these materials under salt stress were analyzed. The main enriched metabolic pathway products were determined by verifying the significantly differentially expressed genes (DEGs). The objective of this article is to clarify the main metabolic processes and differential gene expression patterns involved in the process of improving salt tolerance in tomato seedlings through grafting.

## Materials and methods

### Plant material and treatments

The salt-sensitive tomato variety Zhongza 9 and the strongly salt-tolerant tomato rootstock variety QZ-006 (selected by the research group in the early stage) were used as experimental materials and were purchased from the Vegetable and Flower Institute of the Chinese Academy of Agricultural Sciences (Beijing, CHN) and Beijing Kaixingelin Agricultural Technology Co., Ltd. (Beijing, CHN).

The experiment was conducted at the scientific research base of Hebei Agricultural University. (38° 10 ‘- 40° 00’ N, 113° 40 ‘- 116° 20’ E). When the tomato seedlings developed four leaves and one heart, the split grafting method was used to obtain GSs. We selected GSs and NGS that were uniform and displayed good growth, rinsed the roots, used Hoagland’s nutrient solution formula for hydroponic cultivation, and used an oxygen pump to supplement oxygen to maintain the normal growth of the grafted tomato seedlings for 15 days after grafting. After 3 days of precultivation, the two types of seedlings were subjected to 175 mmol·L^-1^ NaCl, which was added to Hoagland’s nutrient solution. This concentration is the concentration that has significant phenotypic differences in plants selected in previous experiments. (Wu et al., 2020).

In terms of cultivation, the temperature was maintained at 20°C, the pH value of the nutrient solution was maintained at 6.5, and samples were taken at different times after treatment to determine the growth and various physiological indexes of the seedlings. The samples were taken at 8:00 a.m. The leaves of the treated seedlings were harvested at 0 (control), 6, 12, 24, 48 and 96 h for transcriptome sequencing and obtained 12 sets of samples (Three biological replicates for each group of samples, totaling 36 samples). Then the same samples were used for physiological index determination (three biological replicates per treatment). All the samples were immediately placed in liquid nitrogen and stored at -80°C until use.

### Physiological measurements

The grading standard of the salt damage index was the same as that of *Liu* et al. (Liu et al., 2007). The electrolyte leakage rate of tomato seedling leaves was measured by a conductivity meter (Thermo Orion, MA, USA) according to the methods of *Dionisio Sese* and *Tobita* (Dionisio-Sese and Tobita, 1998). The leaves of the seedlings of every sample was added to 5 mL of HNO_3_ (65%~68%) after they were heated in a microwave digestion system for 2~3 hours. The Na^+^ content was determined by inductively coupled plasma-mass spectrometry (ICP-MS; PerkinElmer, Inc., Elan DRC-e) (Chen et al., 2010).

### RNA-seq and data analysis

Frozen leaves of 36 samples were sent to Novogene Bioinformatics Technology Co. (Beijing, China) for RNA-seq. Total RNA was isolated using an RNeasy Mini Kit (Qiagen, Germany) according to the manufacturer’s instructions and then analyzed with 1% agarose gel electrophoresis to determine the RNA integrity and the presence of DNA contaminants. qRT-PCR was used to quantify the effective concentration of the library after the insert size met expectations (the effective concentration of the library was higher than 2 nM) to ensure the quality of the library.

RNA-seq libraries were sequenced on an Illumina HiSeq X Ten platform. After filtering the adapters and low-quality sequences, the clean reads were mapped to the tomato SL 2.0 genome (https://www.ncbi.nlm.nih.gov/genome/7?genome_assembly_id=393272) using HISAT (Kim et al., 2015). DEGs were identified using the DESeq R package (version 1.10.1; http://www.bioconductor.org/packages/release/bioc/html/DESeq.html) based on |log2(fold change)| ≥1 and false discovery rate (FDR) < 0.05.

### qRT-PCR validation

We randomly selected 28 genes for qRT-PCR testing to verify the accuracy of transcriptome. The relative expression level of the candidate genes was analyzed by using the Actin-7 gene as an internal reference, the sequence of which was retrieved from GenBank (GenBank accession number X58253). Gene-specific primers (**Table S1**) were designed; the primers were synthesized by staff at Shanghai Bioengineering Company. qRT-PCR was performed according to the instructions of a Fast Super EvaGreen qPCR Master Mix Kit (US Everbright^®^, Inc.). The experimental results were analyzed by the 2^-ΔΔCT^ method, and each sample included three technical replicates.

### Determination of amino acid contents, photosynthetic indexes and hormone contents

The amino acid contents in the tomato leaves were calculated by the external standard method (Deng et al., 2014). The chlorophyll of the seedlings was extracted with acetone-ethanol (1:1) (Zhang, 1986).

The photosynthetic parameters were measured with an LI-6400 (LI-COR, USA) portable photosynthesis apparatus during sunny days from 9:00 to 11:00 a.m. The potential water use efficiency (*WUEi*) was calculated using the methods of Ashraf et al. (Ashraf, 2003). The chlorophyll fluorescence parameters were measured with a FluorCam open chlorophyll fluorescence imaging system (Czech Republic) on the 3rd leaf from the top of the plants. The chlorophyll fluorescence parameters included the initial fluorescence (*F0*), *Fm* (maximum fluorescence), *Fv/Fm* (maximum photochemical efficiency), actual quantum efficiency of photosystem II (PSII)), photosynthetic electron transfer rate (*ETR*), photochemical quenching coefficient (*qp*), and nonphotochemical quenching coefficient (*NPQ*). The following parameters were calculated (Demmig-Adams et al., 1996; Lang et al., 1996): PSII potential photochemical efficiency (*Fv*/*F0*); antenna conversion efficiency (*Fv’/Fm’*); PSII excitation energy pressure (1-*qp*); chlorophyll fluorescence decay rate (*Rfd*=*(Fm-Fs)/Fs*); and proportion of antenna heat dissipation (*D*=1-*Fv’/Fm’*).

An indirect enzyme-linked immunosorbent assay (ELISA) was used to determine auxin (IAA), gibberellin (GA), cytokinin (ZR), brassinolide (BR), abscisic acid (ABA), jasmonic acid (JA) and salicylic acid (SA) contents, and kit purchased from ThermoFisher Scientific Co., Ltd. (USA). Endogenous ethylene (ETH) was collected, fixed and determined according to the methods of *Ling* (Ling et al., 2008) using a Shimadzu 2010 gas chromatograph equipped with a hydrogen flame ion detector. ETH standard gas was purchased from Sigma Company.

### Statistical analysis

Statistical analysis was carried out by Excel2010 software and SPSS 22.0 software. Duncan’s method was used for data variance analysis and difference significance comparison (P<0.05). The data was expressed as mean ± S.E.M.

## Results

### Physiological investigation of GSs

The salt damage index of the seedlings indicated that the capacity to withstand salinity stress was significantly weaker for the NGSs than for the GSs (**Table 1**). With a salt damage index of 3.33%, NGSs showed symptoms of salt damage at 12 h, but that of the GSs was still 0. Afterwards, the salt damage index of the NGSs was always significantly higher than that of the GSs, reflected by a 40.1%-117.5% improvement from 24-96 h. The electrolyte leakage in the leaves of the NGSs was always significantly higher than that in those of controls and GSs from 12-96 h, suggesting more membrane damage during salt stress in the NGSs than in the GSs (**Table 2**). The Na^+^ content in the leaves increased significantly with prolonged salt stress time (**Table 3**). Compared with the NGSs, the GSs presented significantly lower Na^+^ accumulation, which ranged from 16.4%-35.2%. However, there was no significant difference in the sodium ion content between the two types of seedlings under the normal treatment.

**Table 1:** Salt injury index of grafted and non-grafted tomato seedlings under salt stress.

Type	Treatment	Salt injury index(%)				
		6 h	12 h	24 h	48 h	96 h
Grafted	Control	0	0	0	0	0
	NaCl	0	0	5.67±2.31b	20.33±4.62 b	43.33±2.31 b
Non-Grafted	Control	0	0	0	0	0
	NaCl	0	3.33±2.31 a	12.33±2.31a	36.67±2.31a	60.67±2.31a

**Table 2:** Leaf electrolyte leakage of grafted and non-grafted tomato seedlings under salt stress.

Type	Treatment	Electrolyte leakage(%)				
		6 h	12 h	24 h	48 h	96 h
Grafted	Control	5.33±0.42b	5.85±0.55c	5.92±0.64c	6.11±0.85c	5.24±0.62c
	NaCl	6.05±0.59ab	8.21±0.44b	10.12±1.22b	15.53±1.72b	23.41±2.31b
Non-Grafted	Control	5.24±0.47b	5.38±0.56c	6.13±0.54c	6.26±0.75c	5.87±0.57c
	NaCl	8.11±0.72a	10.43±1.22a	15.27±1.51a	22.37±2.91a	30.25±2.31a

**Table 3:** Na^+^ accumulation in leaves of grafted and non-grafted tomato seedlings under salt stress.

Type	Treatment	Na^+^ content(mg·g^-1^ DW )				
		6 h	12 h	24 h	48 h	96 h
Grafted	Control	6.63±0.25c	6.55±0.24c	6.29±0.35c	6.42±0.21c	6.22±0.22c
	NaCl	7.01±0.23b	8.12±0.30b	8.83±0.38b	9.41±0.27b	10.03±0.41b
Non- Grafted	Control	6.48±0.16c	6.42±0.22c	6.36±0.27c	6.60±0.22c	6.32±0.23c
	NaCl	8.23±0.34a	9.47±0.33a	10.37±0.32a	10.87±0.42a	13.56±0.53a

### Transcriptome analysis under salt stress

**Figure 1:**
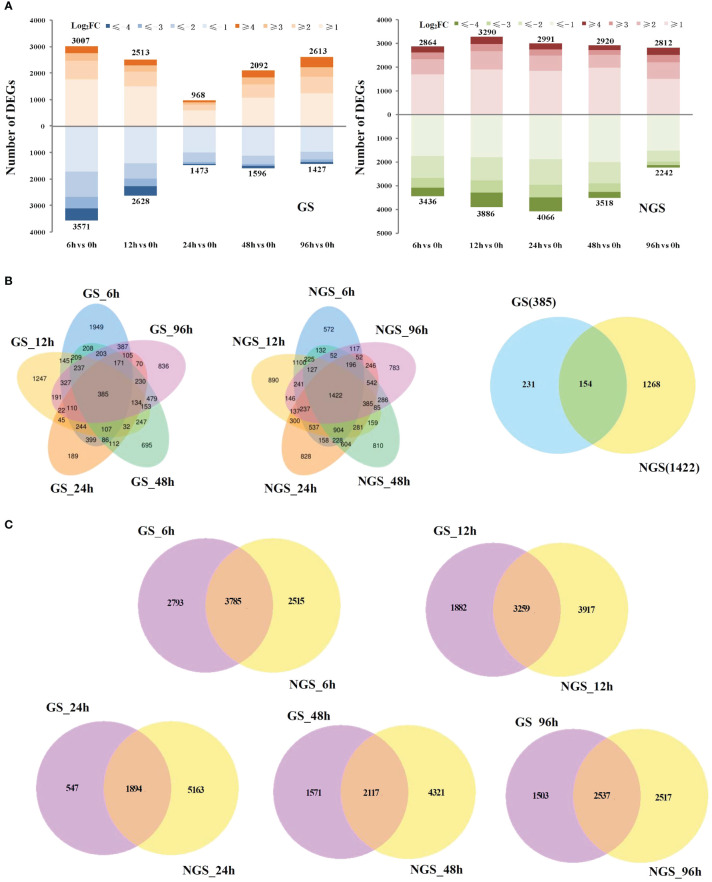
Differentially expressed genes (DEGs) in GS and NGS in response to salt stress. Numbers of DEGs in GS and NGS at different salt stress time points **(A)**. Venn diagrams of DEGs among different salt stress time points in GS and NGS **(B)** and between both genotypes at 6, 12, 24, 48 and 96h **(C)**, respectively.

We obtained ~271 Gb (clean bases) of quality-filtered sequence data (error rate ≤ 0.03) by RNA-seq, and the alignment rate (mapping rate) ranged from 82.53% to 97.26%, with >92.25% of the bases having a quality score of Q30 or higher (**Table S2**). The percentage of reads aligned to the unique location of the tomato genome (unique mapping rate) ranged from 80.63% to 95.58%, and the percentage of reads aligned to the positive and negative chains was higher than 40.30% (**Table S3**). As shown in **Figure S1**, a strong correlation (R^2^>0.92) was observed across all the treatments, which indicated that the biological replicates were reliable in this study. In total, 6578/6300, 5141/7176, 2441/7057, 3688/6438 and 4040/5054 genes were differentially regulated at 6, 12, 24, 48, and 96 h, respectively, compared with the control (0 h) (**Figure 1A**). Venn diagram analysis indicated that many salt-responsive genes were detected at different times under salt stress, which may account for the differences in salt tolerance between the GSs and NGSs (**Figure 1B**). Notably, 154 DEGs were detected at all salt stress time points, which we called ‘core DEGs’. More DEGs were observed at early stress time points, such as 6 and 12 h (**Figure 1C**).

**Figure 2:**
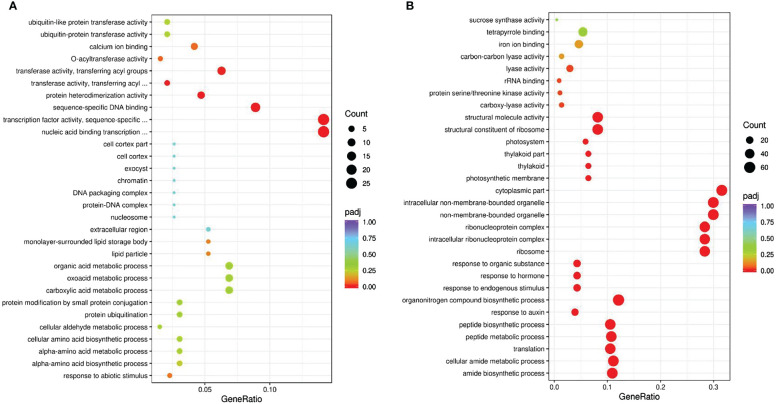
GO enrichment analysis of DEGs in leaves. GS vs GC **(A)** and NGS vs NGC **(B)**.

Several stress-related GO terms were commonly found, such as ‘response to abiotic stimulus’, ‘response to auxin’, ‘response to endogenous stimulus’ and ‘response to hormone’ (**Figure 2A, B**). Gene Ontology (GO) enrichment analysis of the GSs revealed several terms related to amino acid biosynthesis and metabolic processes. There were significant differences in DEG-enriched pathways between the GSs and NGSs at different time points (**Figure S2**). At the initial time of salt stress, the GO terms were enriched in ‘photosynthesis’ at 6 and 12 h. Taken together, these results suggested that the initial stage of the response to salt stress mainly involves several important genes associated with photosynthesis.

**Figure 3:**
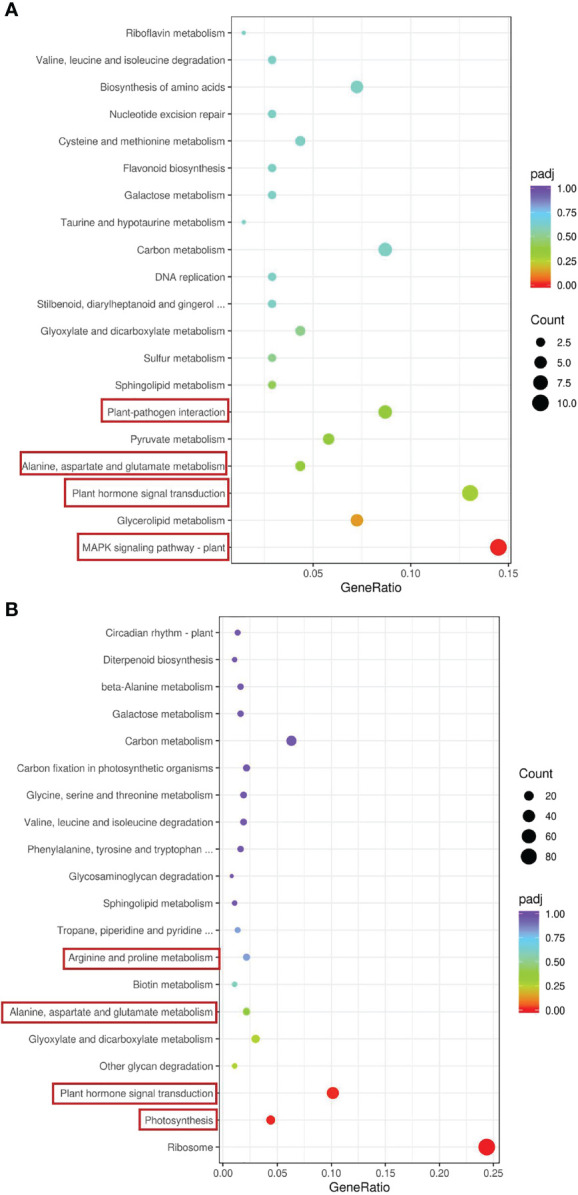
KEGG enrichment analysis of DEGs in leaves. GS vs GC **(A)** and NGS vs NGC **(B)**.The red border indicates that this article focuses on metabolic pathways.

**Figure 4:**
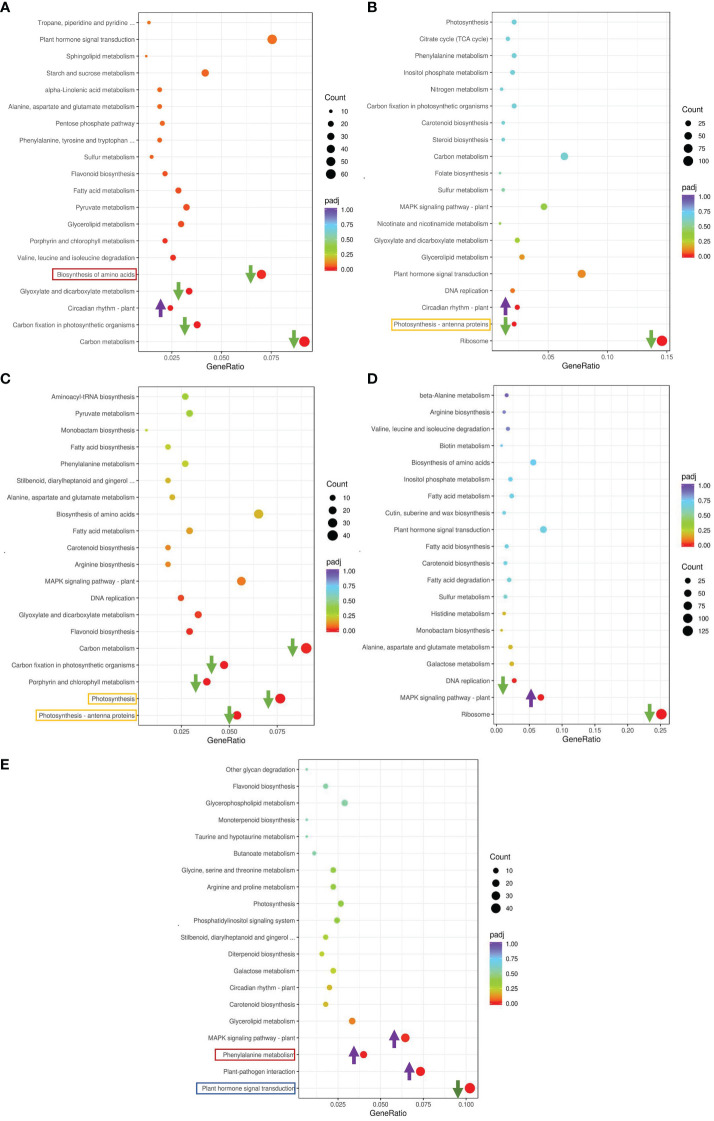
KEGG enrichment analysis of DEGs in leaves. GS_6h vs NGS_6h **(A)**, GS_12h vs NGS_12h **(B)**, GS_24h vs NGS_24h **(C)**, GS_48h vs NGS_48h **(D)**, GS_96h vs NGS_96h **(E)**.

We conducted a Kyoto Encyclopedia of Genes and Genomes (KEGG) enrichment analysis based on the DEGs detected in the GS vs. GC and NGS vs. NGC comparison groups (**Figures 3A, B**). The most significantly enriched pathways of the GSs included ‘MAPK signaling’; ‘plant hormone signal transduction’; ‘alanine, aspartate and glutamate ‘metabolism’; and ‘plant-pathogen interaction’, all of which mainly involve signal transduction and amino acid metabolism. KEGG enrichment analysis of the NGSs also revealed a focus on photosynthesis, in addition to signal transduction and amino acid metabolism. To determine which pathway genes are responsible for the differences in salt tolerance between the two types of seedlings at different stages of salt stress, we analyzed the KEGG pathways at each time point. The significantly enriched pathways at each time point were evaluated. Under salt stress, most of these pathway genes were downregulated at each time point. The DEGs upregulated at 6 h and 12 h were significantly enriched in ‘circadian rhythm - plant’, while the DEGs downregulated at 6 h were enriched in ‘carbon metabolism’, ‘biosynthesis of amino acids’, and ‘carbon fixation in photosynthetic organisms’, where the DEGs enriched in ‘ribosome’ and ‘photosynthesis - antenna proteins’ at 12 h were downregulated (**Figures 4A, B**). The genes differentially expressed at 24 h were enriched in ‘photosynthesis - antenna proteins’, ‘photosynthesis’, ‘porphyrin and chlorophyll metabolism’, etc., all of which are related to photosynthesis and were downregulated (**Figure 4C**). At 48 h, DEGs enriched in ‘ribosome’ and ‘DNA replication’ pathways were downregulated, and those enriched in the ‘MAPK signaling pathway-plant’ were upregulated (**Figure 4D**). At 96 h, there were three pathways whose enriched DEGs were upregulated: ‘plant-pathogen interaction’, ‘phenylalanine metabolism’ and ‘MAPK signaling pathway-plant’, while those enriched in ‘plant hormone signal transduction’ were downregulated (**Figure 4E**).

**Figure 5:**
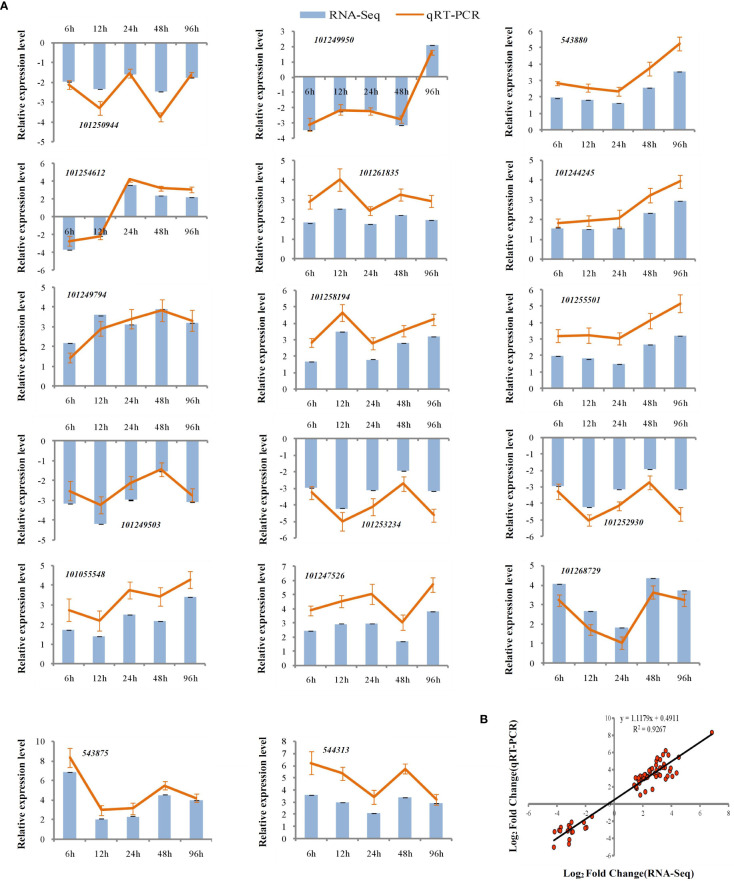
qRT-PCR validation of expression profiles obtained by RNA-seq in GS under salt stress. qRT-PCR analysis of 17 selected genes. The Y-axis represents the relative expression levels, the X-axis shows the time of 175 mM NaCl treatment in GS **(A)**. The correlation of log_2_ Fold Change obtained by qRT-PCR (Y-axis) and RNA-seq (X-axis) in GS **(B)**.

**Figure 6:**
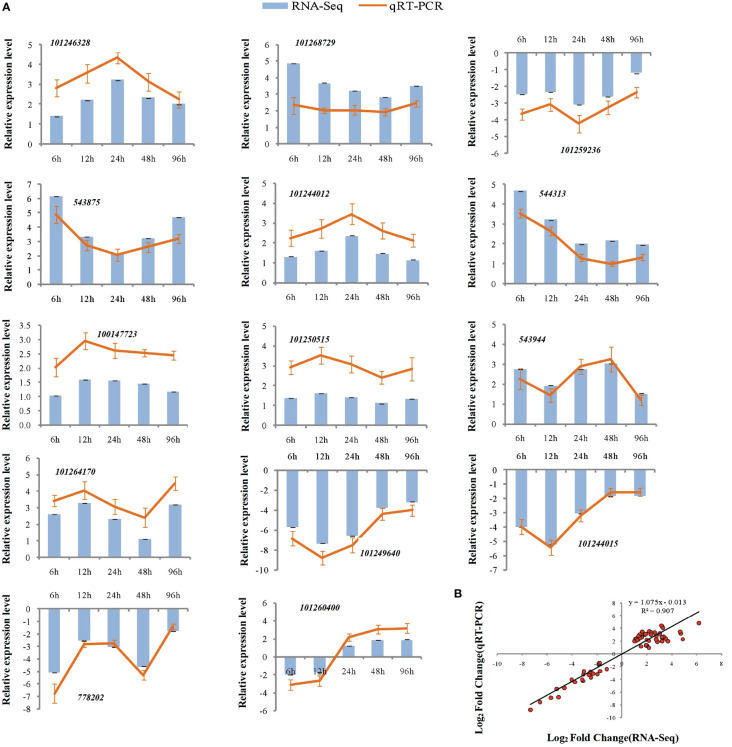
qRT-PCR validation of expression profiles obtained by RNA-seq in NGS under salt stress. qRT-PCR analysis of 14 selected genes. The Y-axis represents the relative expression levels, the X-axis shows the time of 175 mM NaCl treatment in NGS **(A)**. The correlation of log2 Fold Change obtained by qRT-PCR (Y-axis) and RNA-seq (X-axis) in NGS **(B)**.

**Figure 7:**
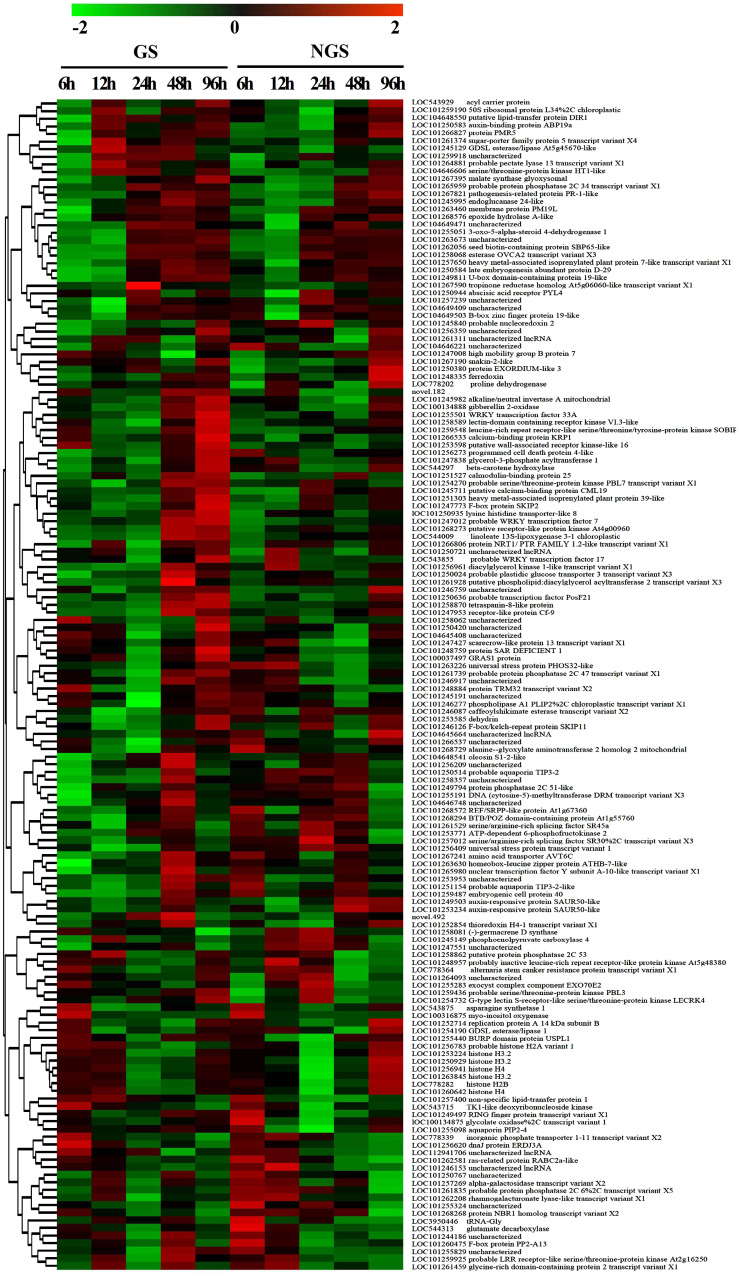
Heatmap of analysis of 154 core DEGs in GS and NGS at different salt stress time points. X-axis, samples; Y-axis, differentially expressed gene names with annotations.

To assess the reliability of the RNA-seq data, qPCR was used to analyze the expression patterns of 31 randomly selected genes (**Figures 5A**, **6A**). The correlation coefficients (R^2 = ^0.9267, R^2 = ^0.9072) implied that the RNA-seq data were reliable (**Figures 5B**, **6B**). The temporal and spatial expression patterns of 154 core DEGs are presented in a heatmap (**Figure 7**). There were 4 transcription factor-encoding genes among the 154 core genes coenriched within the two types of seedlings under salt stress in our study, including *WRKY* (LOC101255501, LOC101247012, LOC543855) transcription factors and the basic leucine zipper type (*bZIP*) transcription factor *PosF21* (LOC101250636). Moreover, in terms of expression level, these four genes in the GSs exhibited increased expression most of the time after salt stress, suggesting that they play important roles in transcriptional regulation that contributes to the salt tolerance of tomato. Other DEGs exhibited similar expression patterns, indicating that these genes were commonly induced in this species in response to salinity stress.

### Amino acid contents and related gene expression levels

**Figure 8:**
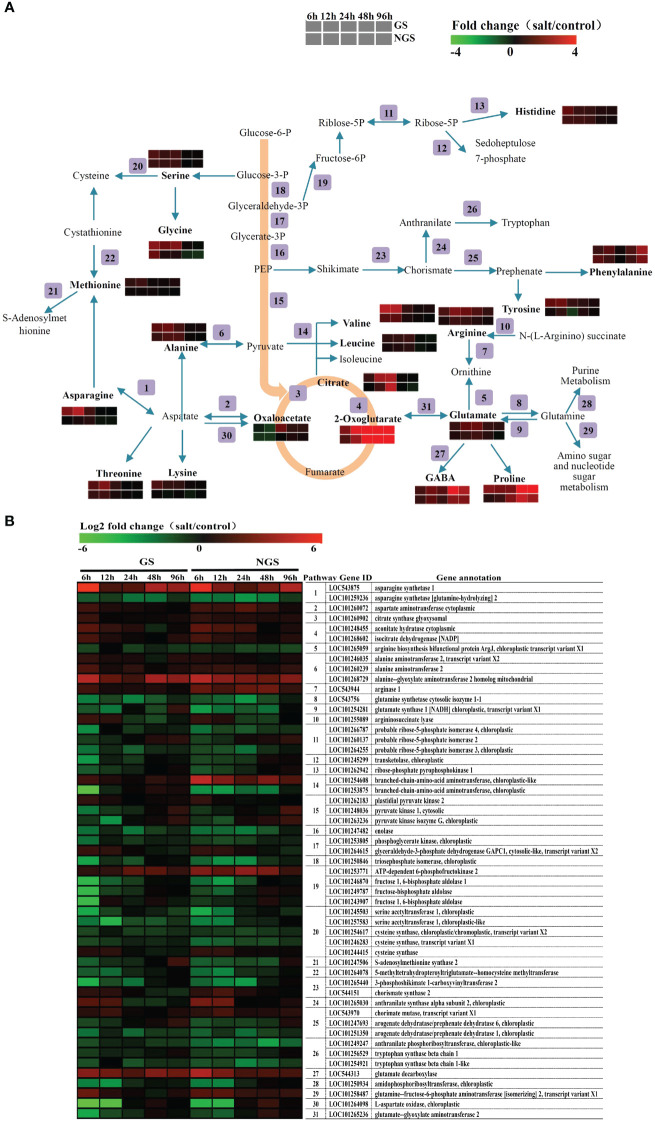
Adaptive changes in amino acid metabolism in GS and NGS during salinity stress. Amino acid metabolic pathways and the general pattern of metabolites changes associated with the pathways in GS and NGS at 6 h to 96 h after salinity stress **(A)**. Expression changes of the genes involved in metabolic pathways in GS and NGS at different salt stress time points. The pathway ID column indicates the gene product in relation to metabolic pathway **(B)**.

On the basis of the results of the KEGG pathway enrichment analysis, which revealed enrichment of amino acid-related pathways at 6 h and 96 h, we carried out a detailed analysis of amino acid metabolism pathway genes. **Figure 8** shows the amino acid biosynthesis pathways and the general pattern of the relative changes in related metabolites in the GS and NGS genotypes under salt stress conditions. The content of most free amino acids considered osmotic regulatory substances significantly increased in both genotypes, implying that the accumulation of free amino acids is crucial in the response to salt stress. Obviously, the accumulation rate of many free amino acids and related compounds (including alanine, asparagine, glutamate, glycine, leucine, lysine, methionine, phenylalanine, proline, γ-aminobutyrate (GABA), serine, threonine, tyrosine, and valine) was higher in the GSs than in the NGSs, which may be a positive feature for withstanding salinity stress by the GS genotype. The change in most free amino acids was detected mainly from 6-24 h, but the contents of proline and GABA, which are major stress-related amino acids, changed markedly from 48-96 h.

The expression of genes related to the amino acid metabolism pathway also changed significantly, as shown in **Figure 8B**. Most of the genes involved in the biosynthesis of these amino acids and related compounds, such as asparagine, aspartate, alanine, arginase and GABA, were upregulated in both genotypes under salt stress. At the same time, there was also a trend towards downregulation of several genes that encode related enzymes, such as those involved in the arginine, glutamate, and cysteine pathways, but most of these genes did not encode synthetases. Several genes, such as those encoding the arginine biosynthesis bifunctional protein *ArgJ* (LOC101265059), glutamate synthase 1 (NADH; chloroplastic; LOC101254281), and S-adenosylmethionine synthase 2 (LOC101247506), showed higher expression changes in the GSs than in the NGSs at 12 h to 48 h after salt stress. In addition to those involving amino acids, genes and products related to organic acid metabolism, especially that of citrate and 2-oxoglutarate, were also significantly upregulated. Moreover, the expression of the gene encoding glutamate decarboxylase (LOC544313), which is involved in the GABA biosynthesis pathway, was upregulated markedly, explaining the rise in GABA levels. Overall, the integration of the two omics datasets showed that amino acid metabolism strongly promoted the salt tolerance of tomato, especially in the GS genotype.

### Photosynthesis and related gene expression levels during salinity stress

**Figure 9:**
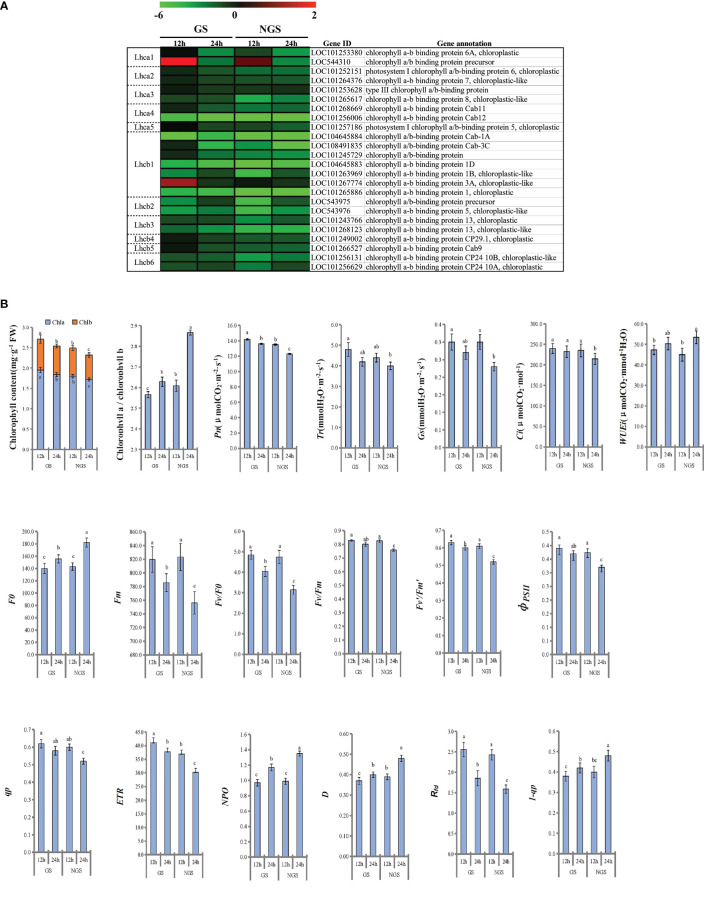
Photosynthetic antenna protein related genes of leaves at 12 h and 24 h after salinity stress **(A)**. Photosynthesis and fluorescence related parameters of leaves at 12 h and 24 h after salinity stress **(B)**.

Considering that photosynthesis has been shown to play vital roles in the abiotic stress response in the initial stage and middle stage of salt stress, we focused on 24 DEGs enriched in the ‘photosynthesis - antenna proteins’ pathway (**Figure 9A**). In general, the expression of the genes encoding antenna pigment proteins showed a general downwards trend after salt stress. Notably, the gene expression in the GS genotype was higher than that in the NGS genotype, especially at 12 h, which may be a positive feature for withstanding salinity stress in the GS genotype. LHCI is the major light-trapping pigment protein of photosystem I (PSI). The expression levels of the genes encoding chlorophyll a-b binding protein 6A (LOC101253380) and the chlorophyll a/b binding protein precursor (LOC544310) in the GSs were significantly higher than those in the NGSs at 12 h. *Lhcb1* is one of the genes encoding chlorophyll a/b binding protein in PSII. The gene encoding chlorophyll a/b binding protein 3A (LOC101267774) showed an increase in expression in the GSs at 12 h. At 24 h of stress, all photosynthetic antenna pigment protein pathway-related genes were significantly downregulated in both the GSs and the NGSs.

We calculated the related photosynthesis indexes and found that the photosynthesis of the seedlings of both genotypes was inhibited under salt stress (**Figure 9B**). However, compared with the NGSs, the GSs presented better photosynthesis-related indexes. The chlorophyll content in the GSs was significantly higher than that in the NGSs, and the chlorophyll a/b ratio was significantly lower in the GSs than in the NGSs, especially at 24 h. The *Pn*, *Tr*, *Gs* and *Ci* of the tomato seedlings decreased continuously with the prolongation of NaCl stress. The *Pn* and *Ci* of the GSs were significantly higher than those of the NGS at 24 h. With prolonged NaCl stress time, the chlorophyll fluorescence parameters of both genotypes showed a downwards trend. The parameters of the GSs, such as *F0*, *Fm*, *Fv/F0*, *Fv/Fm*, *Fv’/Fm’*, *φPSII*, *qp*, *ETR* and *1-qp*, were significantly higher than those of the NGSs. However, the *NPQ*, *D* and ***R_fd_*** of the GSs were significantly lower than those of the NGSs.

### Hormone signal transduction pathway DEGs and hormone contents during salinity stress

**Figure 10:**
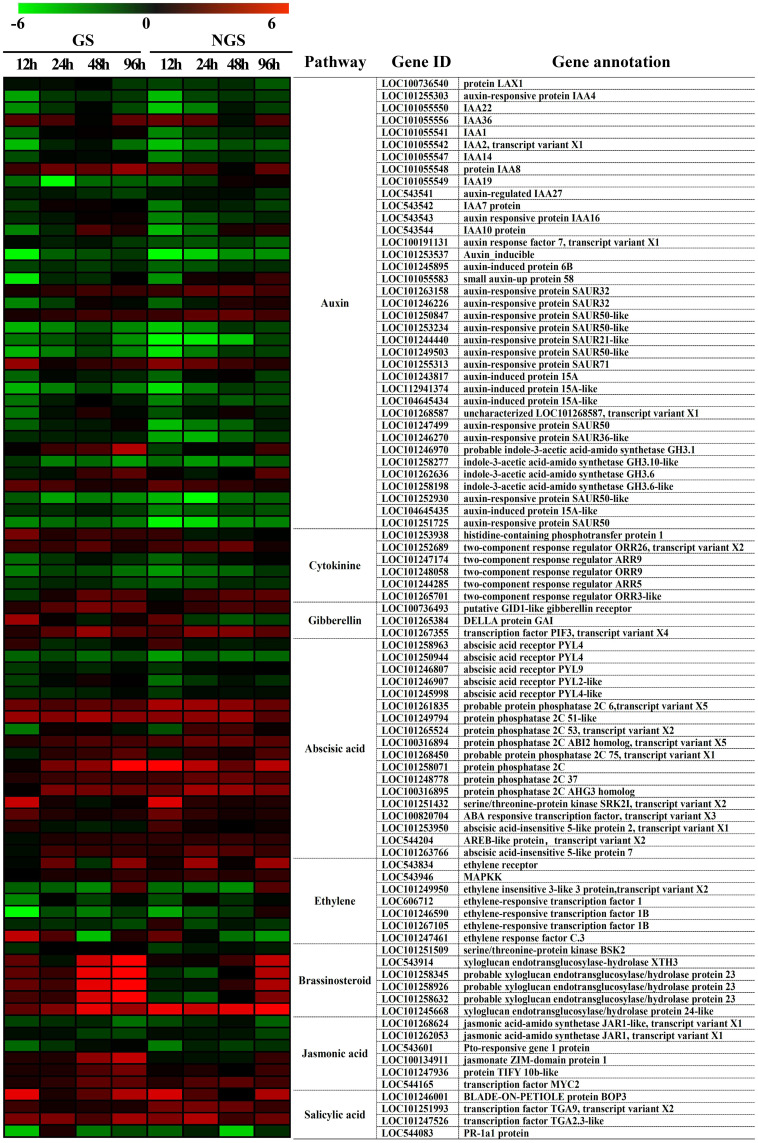
Heatmap of genes involved in hormone signal transduction in GS and NGS at 12 h-96 h after salinity stress. X-axis, samples; Y-axis, differentially expressed gene names with annotations.

The ‘plant hormone signal transduction’ KEGG pathway was shown to be enriched in DEGs during almost the whole stress process, and plant hormones have been shown to play vital roles in the abiotic stress response. We focused on 87 DEGs enriched in the ‘plant hormone signal transduction’ pathway from 12-96 h (**Figure 10**). The expression of DEGs in the two genotypes was different. In particular, several genes involved in the IAA, ZR, GA, brassinosteroid, and JA pathways, such as those encoding probable indole-3-acetic acid-amido synthetase *GH3.1* (LOC101246970), histidine-containing phosphotransfer protein 1 (LOC101253938), DELLA protein GAI (LOC101265384), xyloglucan endotransglucosylase-hydrolase *XTH3* (LOC543914), probable xyloglucan endotransglucosylase/hydrolase protein 23 (LOC101258345, LOC101258926 and LOC101258632), jasmonate ZIM-domain protein 1 (LOC100134911) and protein TIFY 10b-like (LOC101247936), showed higher levels in the GSs than in the NGSs after exposure to salt. Moreover, the difference in BR signaling pathway genes between the two materials was the most significant. These genes are mainly involved in promoting beneficial hormones involved in stress resistance, which together may be one of the reasons for the increased salt tolerance of the GSs.

**Figure 11:**
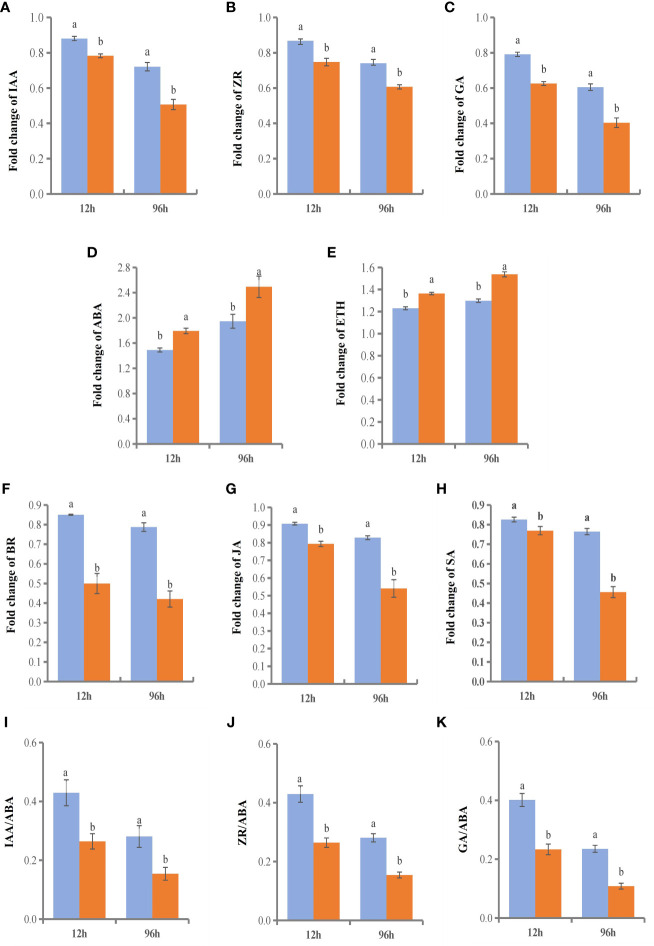
Hormone content and ratio of leaves in GS and NGS at 12 h and 96 h after salinity stress. A-H represents levels of different hormone content. I-K represents the ratio of different hormones.

We selected two time points with significant gene enrichment to determine leaf hormone contents (**Figure 11**). The hormone change range in the NGSs was larger than that in the GSs. Between the two genotypes, the contents of IAA, ZR, gibberellic acid (GA), BR, methyl jasmonate (MeJA) and SA, which are considered to be positively related to stress tolerance, showed a downwards trend. However, the time points at which the various hormones in the GSs occurred sooner than those in the NGSs. Among them, the BR content decrease of NGSs was greater than that of other hormones, with more than 50%. However, the BR content of GSs decreased by only 15% and 21.3%. The ABA and ETH contents increased, and the increases in the GSs were less drastic than they were in the NGSs. In addition, salt stress also affected the hormone balance in the two types of seedlings. The ratios of IAA, ZR and GA to ABA also decreased significantly in both genotypes, but these decreases were still greater in the NGSs than in the GSs.

## Discussion

### Salt-tolerant rootstock grafting inhibits the accumulation of Na^+^ in the leaves

The damage to plant cells and the resulting growth inhibition under salt stress are mainly caused by the excessive absorption and accumulation of Na^+^ (Flowers and Colmer, 2015). Under salt stress, the absorption of Na^+^ by plant roots is significantly increased. Na^+^ is translocated from the roots and stems to the leaves, causing ion toxicity in leaf cells and osmotic stress, which disrupts the structure and function of the membrane system (Tian et al., 2012), causing plants to display obvious salt stress symptoms. The salt tolerance of nonhalophytes mainly depends on the ability of their root system to selectively absorb salt ions and control the transport of those ions into the roots from the soil (Yang et al., 2015). Our previous study showed that the growth potential of GSs is significantly different due to different rootstocks and that the salt tolerance of GSs is mainly determined by the resistance of the root systems of rootstocks (Jia et al., 2020).

The results of the present experiments show that salt stress leads to a gradual increase in Na^+^ content in the two types of tomato seedlings. The Na^+^ content in the leaves of GSs was found to be significantly lower than that in the leaves of NGSs, and the salt stress index of the leaves of NGSs was higher than that of the leaves of GSs. This is because salt-tolerant rootstocks have a strong ability to avoid and reject sodium. Less Na^+^ is absorbed by salt-tolerant roots than by salt-sensitive roots, and more Na^+^ is trapped in the roots to reduce the transport of Na^+^ to the leaves, alleviating the damage caused by salt stress to seedlings. However, which metabolic processes related to the salt stress response are accompanied by rootstocks inhibiting the accumulation of sodium ions on the leaves, thus alleviating the salt stress damage on the leaves?

### GSs stabilize the number of genes expressed and have different expression modes

With the development of molecular biology technology, it has been found that there is a wide range of genetic material exchange and interactions that occur in grafts, including those involving mRNAs, sRNAs, proteins and so forth (Lewsey et al., 2016; Lu et al., 2020; Wang et al., 2020). Transcriptome sequencing of disease-resistant and nondisease-resistant rootstocks in tomato showed that the former were less sensitive to virus-induced injury (Spanò et al., 2020). In the present study, the total number of DEGs in the GSs was significantly lower than that in the NGSs throughout the whole process of salt stress (**Figure 2A**), with ratios equal to 0.72, 0.35, 0.57 and 0.80. Moreover, the variation trend of the expression of DEGs in the GSs and NGSs at the different time points was also different. The number of DEGs in the GSs was highest at 6 h and decreased to the lowest value at 24 h. However, the number of DEGs in the NGSs decreased gradually after peaking at both 12 h and 24 h. These results indicate that the GS gene response was faster than the NGS gene response and that maintaining the stability of gene expression may underpin the molecular basis for inducing salt tolerance in GSs with salt-tolerant rootstocks.

Based on the phenotypic and physiological data, we considered the time spanning 6 and 12 h as the initial stage of salt stress, the time spanning 24 h and 48 h as the middle phase of salt stress, and 96 h as the later stage of salt stress. Generally, the change trend of the expression of DEGs in the two types of seedlings at each time point was mainly downwards, especially in the early and middle stages of stress. Photosynthetic pathway gene enrichment mainly occurred during the above two periods. Amino acid metabolic pathways were mainly enriched in the early and late stages of stress, while hormone signal transduction was enriched throughout the whole process of stress. These three pathways are closely related to salt stress, and further studies are necessary.

Previous studies have shown that many important salt tolerance-related genes are directly regulated by transcription factors related to the stress response. *ABI4* in Arabidopsis regulates the expression of the *HKT1* gene (Shkolnik-Inbar et al., 2013). *NAC* transcription factors are induced in response to salt stress and participate in the regulation of salt tolerance-related genes in the root system of pumpkin rootstocks (Cao et al., 2017). The *WRKY* transcription factor family is one of the largest transcription factor families in plants. *WRKY* members participate in the plant response to biotic/abiotic stress, growth and development and are important components of the plant regulatory network (Xu et al., 2016). The three *WRKY* genes selected in this study were *WRKY33A, WRKY7* and *WRKY17. AtWRKY33* controls the formation of an extracellular barrier in Arabidopsis roots by regulating the cytochrome P450 gene, thus improving salt tolerance (Krishnamurthy et al., 2020). *WRKY7* may be involved in the response of rice to bacterial blight (Zheng, 2018), and *WRKY17* regulates osmotic stress and ABA-induced stomatal closure. In addition, *PosF21* plays a positive regulatory role in the ABA signaling pathway, which may be related to salt stress tolerance (Zhong, 2016). Therefore, we speculate that the abovementioned transcription factors are important, and the regulatory mechanism needs to be further verified.

### Leaves of GSs accumulate more amino acids

In addition to inhibiting plant growth, salt stress also causes plants to initiate metabolic changes, in which the osmotic regulation of amino acids plays an important role in improving plant salt tolerance (Zhang et al., 2017). An increase in amino acid content can also improve the adaptability of plants to salt stress. Previous studies have shown that amino acid synthesis is one of the adaptive responses of plants to NaCl stress (Misra and Gupta, 2005). Moreover, previous studies have mostly focused on the relationships between salt stress and total proline and free amino acid contents. The salt tolerance of grafted tomato seedlings can be improved by inducing an increase in the free proline content (Chen et al., 2005). Free amino acids may be important osmotic pressure regulators in the cytoplasm of plant cells, perhaps due to the accumulation of toxic $\text{N}{\text{H}}_{4}^{+}$ in plants under salt stress, and it is possible to increase the tolerance of some plants to salt stress (Xu et al., 2016).

In this study, the contents of 16 main amino acids and 3 metabolic intermediates involved in the tricarboxylic acid (TCA) cycle in the leaves of tomato seedlings under salt stress were analyzed. Although the contents of most amino acids increased, their peak accumulation occurred mainly in the early stage of stress but then decreased slowly. However, in contrast to the KEGG data, this increase in amino acid content continued from the initial stage to the late stage of salt stress. Compared with those of the other amino acids and related compounds, the degree of increase and duration of proline and GABA were higher and longer, which played an important role in alleviating salt stress injury in the grafted tomato seedlings. Moreover, glutamate, which is the precursor of proline and GABA and is an important intermediate of carbon and energy metabolism (Dent et al., 1947), also presented sustained accumulation. However, at the gene level, the two genotypes showed no similar change trend. Only for a few biosynthesis-related genes was the expression level in the GSs higher than that in the NGSs. We speculate that in addition to the increase in the expression level of enzymes related to the amino acid synthesis pathway, GSs may employ other mechanisms to regulate amino acid contents *in vivo*, for example, inhibiting the decomposition of amino acids. The significant increase in multiple amino acids in GS leaves should be the result of improved salt tolerance by grafting. Specifically, it has stronger osmoregulation ability and improves the tolerance to sodium ion stress.

### Photosynthesis of GSs remained relatively stable

In higher plants, PSI and PSII, which are the two different light energy conversion systems on the thylakoid membranes, have their own light harvesting pigment protein complexes. The complex associated with PSI is the light harvesting pigment protein complex LHCI, which is composed of various LHCA antenna protein subunits (Huang et al., 2018). The complex associated with PSII is the light harvesting pigment protein complex LHCII, which is composed of various LHCB antenna protein subunits. LHCII is the most important pigment protein complex; LHCII accounts for nearly 50% of the pigment total content and approximately 1/3 of the protein embedded in the photosynthetic membranes. LHCII plays an important role in the capture, transmission and transformation of light energy (Ruban et al., 2022).

Our research revealed that both types of tomato seedlings presented decreases in chlorophyll content and photosynthetic indicators during the early and middle stages of stress, but the inhibition of photosynthesis of the NGSs was significantly greater than that of the GSs. By comparing the expression of genes involved in the photosynthesis - antenna protein pathway, we found that the expression in the GSs remained relatively stable at 12 hours compared with that of the NGSs, even if the gene level was downregulated. Lhca1 and Lhcb1, the main light harvesting chlorophyll a/b binding proteins composing the photosystems, are the most abundant members of the light harvesting antenna family and play important roles in the capture, transmission and transformation of light energy. We observed that the two genes (chlorophyll A-B binding protein 3A and chlorophyll a/b binding protein precursor) encoding these proteins were upregulated at 12 h, and the expression level in the GSs was significantly higher than that in the NGSs, partly alleviating the damage to PSI and PSII caused by salt stress. We believe that the difference in the photosynthetic response of the two types of seedlings under salt stress is caused by the different salt tolerances of the roots. In addition to that of antenna proteins, maintenance of the homeostasis of the proteins active in photosynthesis in GSs must also rely on the joint action of other relevant genes, and key genes need to be further identified.

### GSs maintain the content of growth-promoting hormones, and some transcription factors may play an important role

Plant hormones are organic trace substances that accumulate in response to specific environmental signals, and at low concentrations, they can regulate plant physiological reactions (Yu et al., 2020). It has been reported that many hormones are involved in the molecular signaling response to salt stress. Plant hormones are classified according to their function in plant growth and in response to stress, with IAA, ZR, GA, BR, and strigolactones (SLs) classified as growth-promoting hormones and ABA, SA, JA and ETH considered stress response hormones (Verma et al., 2016; Shu et al., 2022). Growth-promoting hormones play important roles in maintaining plant growth under salt stress, and ABA and other signaling compounds are also important in the response to stress, as these play roles mainly through signal transduction pathways (Yang and Guo, 2018). We found that the changes in the contents of various hormones under salt stress were in accordance with reported regulatory patterns, with a higher content of growth-promoting hormones in the GSs than in the NGSs and lower ABA and ETH contents. This difference in hormone contents is also one of the reasons why the phenotype of the GSs under salt stress is better than that of the NGSs. Notably, 8 transcription factors were identified that are involved in the GA, ABA, SA, JA and ETH pathways. The expression of transcription factor-encoding genes involved in all of these pathways was significantly upregulated except in the case of ETH-related transcription factors.

Previous studies on hormone responses to salt stress have focused on the ABA signaling pathway (Verslues and Zhu, 2004). There was a significant difference in the expression patterns of different genes involved in the BR pathway between GSs and NGSs in our study, with the xyloglucan endotransglucosylase-hydrolase *XTH3* (LOC543914) and probable xyloglucan endotransglucosylase/hydrolase protein 23 (LOC101258345, LOC101258926, LOC101258632) expression levels in the GSs being significantly higher than those in the NGSs. *Xths* acts on the microfilament-matrix interface during cell elongation (Vissenberg et al., 2005a; Vissenberg et al., 2005b), maintaining the thickness, integrity and strength of the cell wall (Vissenberg et al., 2001; Rose et al., 2002; Jimenez et al., 2006). In *Arabidopsis thaliana*, *XTH* affects the growth of the main roots and stems and participates in light morphogenesis; gene expression is regulated by many plant hormones. The expression of *AtXTH3* and *AtXTH23* is induced by BR signaling (Keuskamp et al., 2011; Song et al., 2018). There are 25 *XTH* genes in the tomato genome. Previous studies on this topic have mainly focused on pectin metabolism (Muñoz-Bertomeu et al., 2013; Tsuchiya et al., 2015); however, little attention has been given to its relation to stress. The results of our study suggested that the upregulation of several *Xth* genes in the leaves of grafted tomato seedlings increased BR signal transduction. These findings provide a new idea for the mechanism underlying the salt tolerance of grafted tomato.

## Conclusion

**Figure 12:**
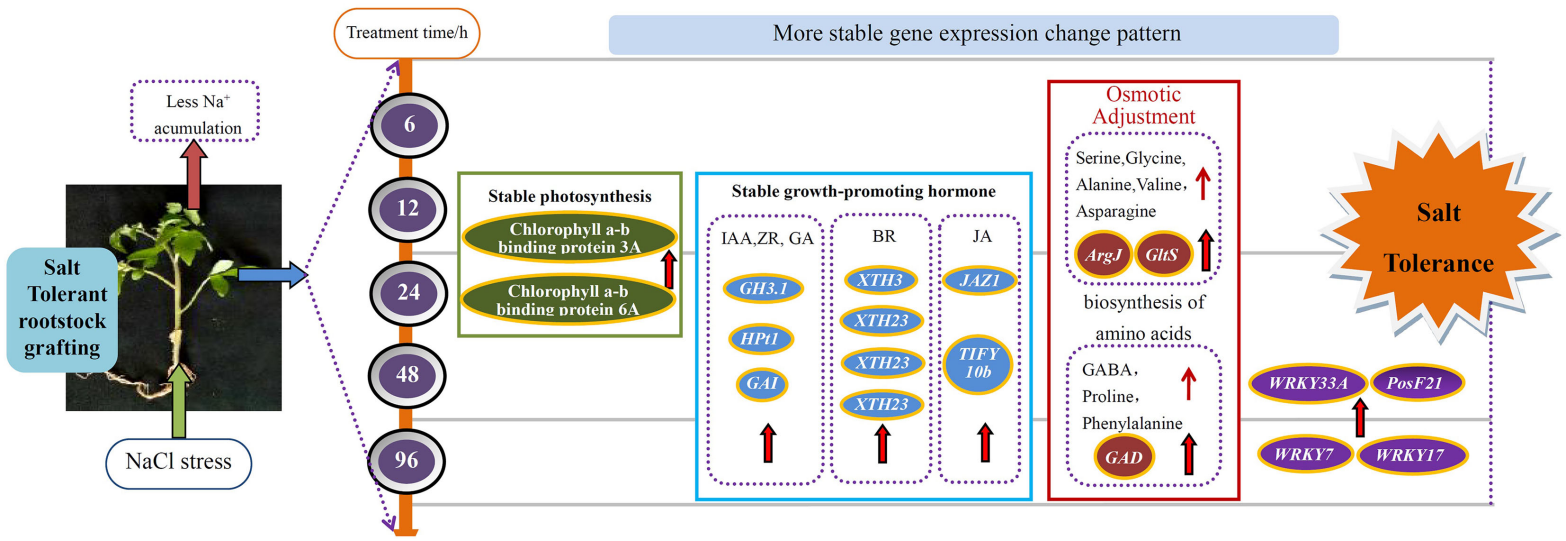
A model for mechanisms underlying the enhanced salt tolerance of grafted tomato.

In summary, the leaves of tomato seedlings grafted with salt tolerant rootstocks obtained higher salt tolerance than before grafting. At the transcriptional level, GS leaves showed a more stable pattern of gene expression changes under salt stress. The differences in leaf genes between the two types of seedlings mainly focus on pathways such as amino acid synthesis, photosynthetic antenna proteins, and hormone signal transduction. Among them, transcription factors such as WRKY, PosF21, and XTH are the most significant in the BR signaling pathway. The tolerance of grafted seedlings to salt stress may be due to the synergistic effect of the entire regulatory network, including the physiological regulatory mechanisms, amino acids, various TFs, and hormones of grafted seedlings of tomato salt tolerant rootstocks (**Figure 12**). It is certain that grafting on salt tolerant rootstocks can bring different metabolic processes and transcriptional level changes to the scion leaves, thereby making the scion leaves show stronger salt tolerance. Further comprehensive research is needed to further verify the role of key genes in metabolism.

## Data availability statement

The data presented in the study are deposited in NCBI, accession number GSE233233.

## Author contributions

XW and HG conceived and designed the research. XW, XB, RH, and DY conducted the experiments. GL, BG, JL, and SL contributed new reagents and analytical tools. All authors contributed to the article and approved the submitted version.
