# Supplementary figures and images for: Transcriptome analysis showed that tomato-rootstock enhanced salt tolerance of grafted seedlings was accompanied by multiple metabolic processes and gene differences
Source: Front Plant Sci. 2023 Jun 2;14:1167145. doi: 10.3389/fpls.2023.1167145 (PMC10272605; doi:10.3389/fpls.2023.1167145)

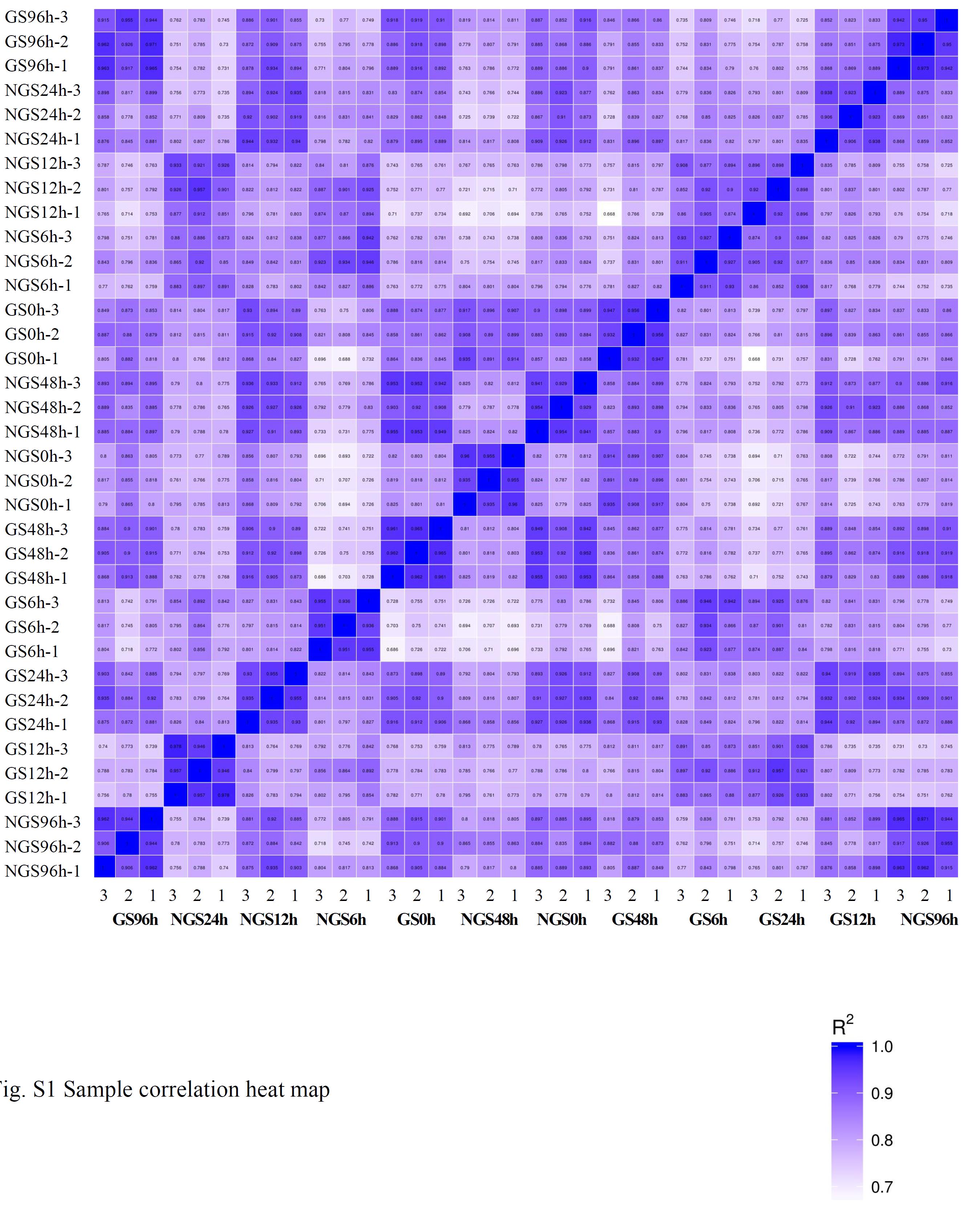

Supplement: Supplementary file 1 [file DataSheet_1.zip › additional file/FIG S1.jpg]

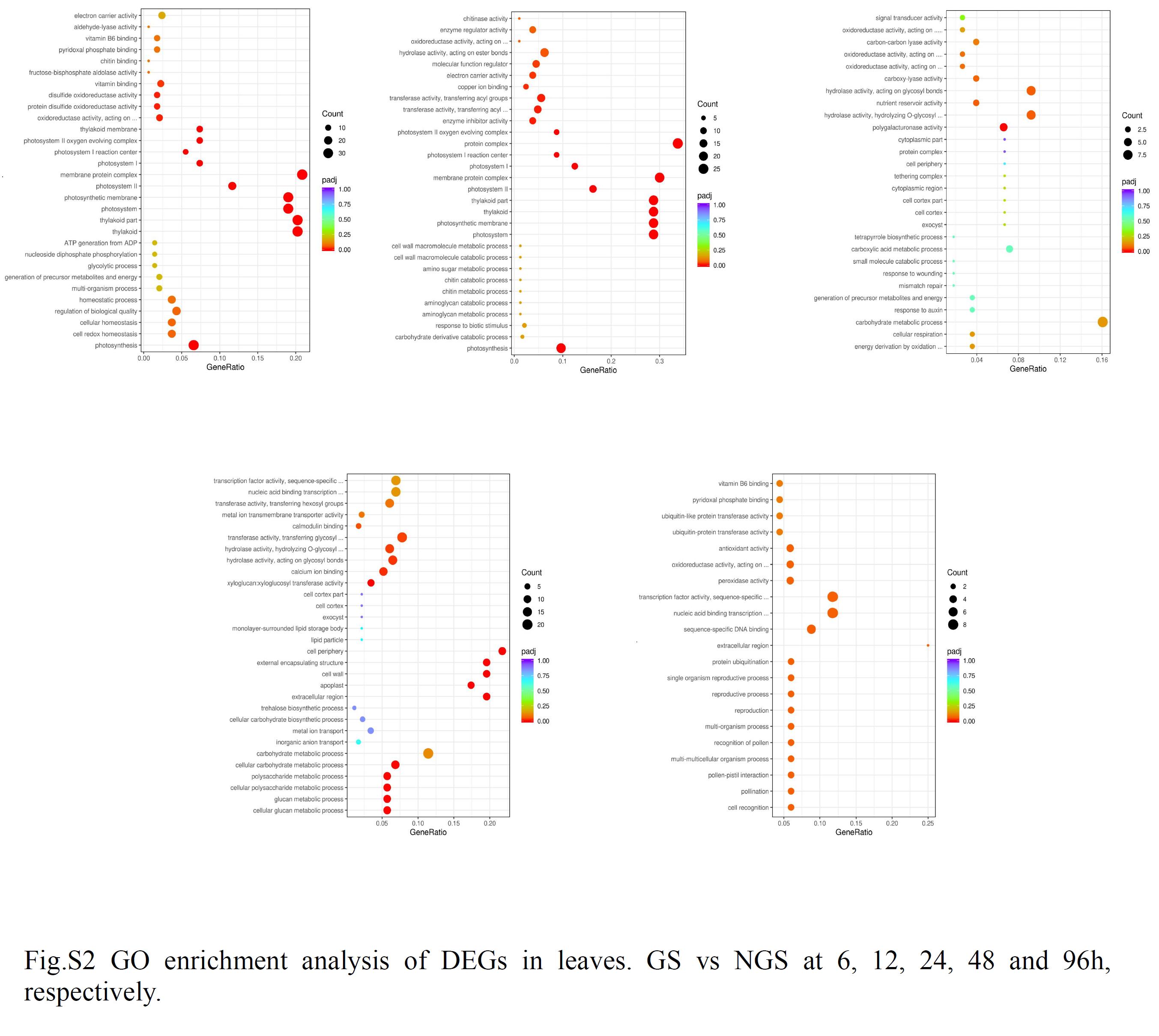

Supplement: Supplementary file 1 [file DataSheet_1.zip › additional file/FIG S2.jpg]

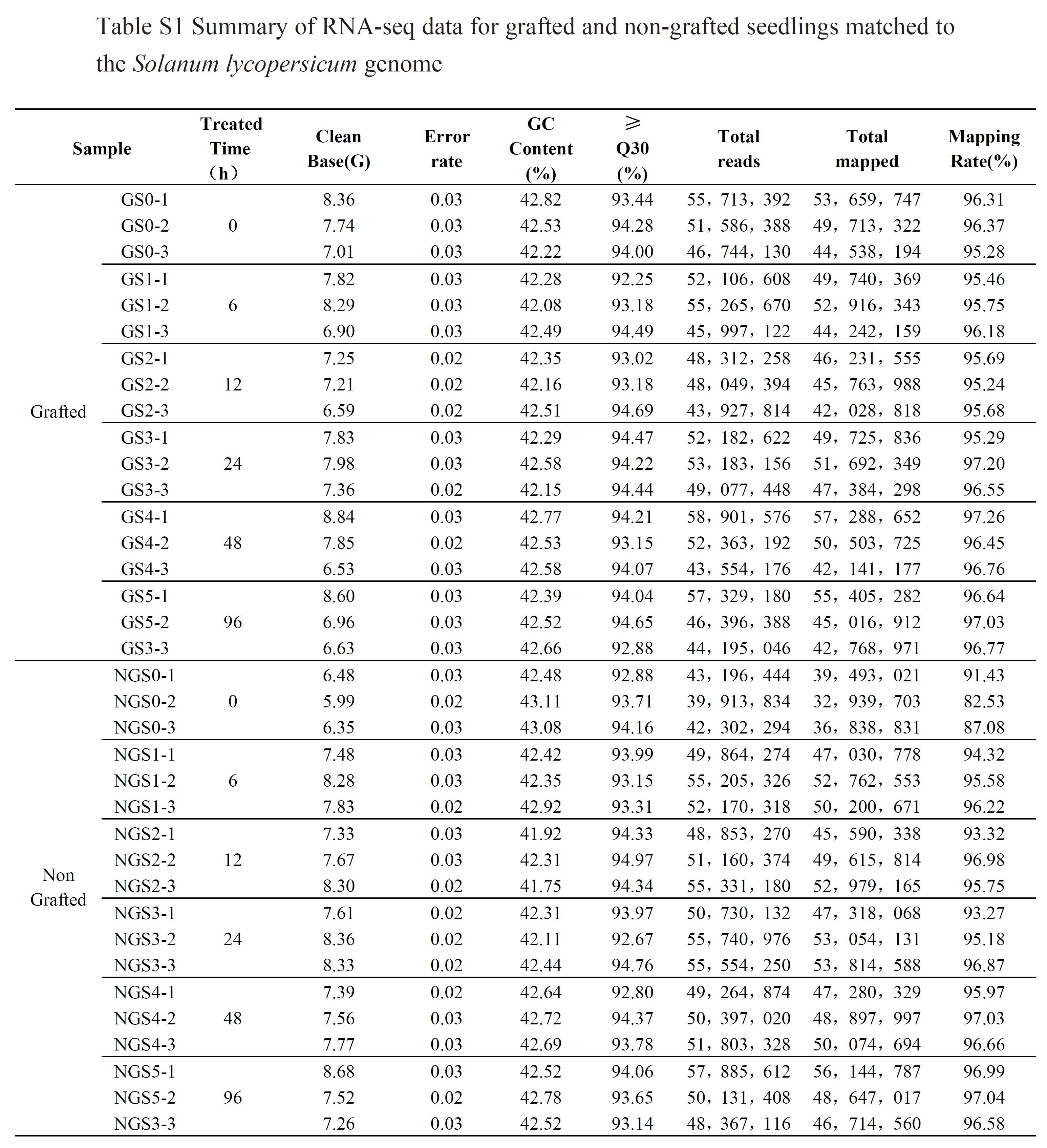

Supplement: Supplementary file 1 [file DataSheet_1.zip › additional file/Table S1.jpg]

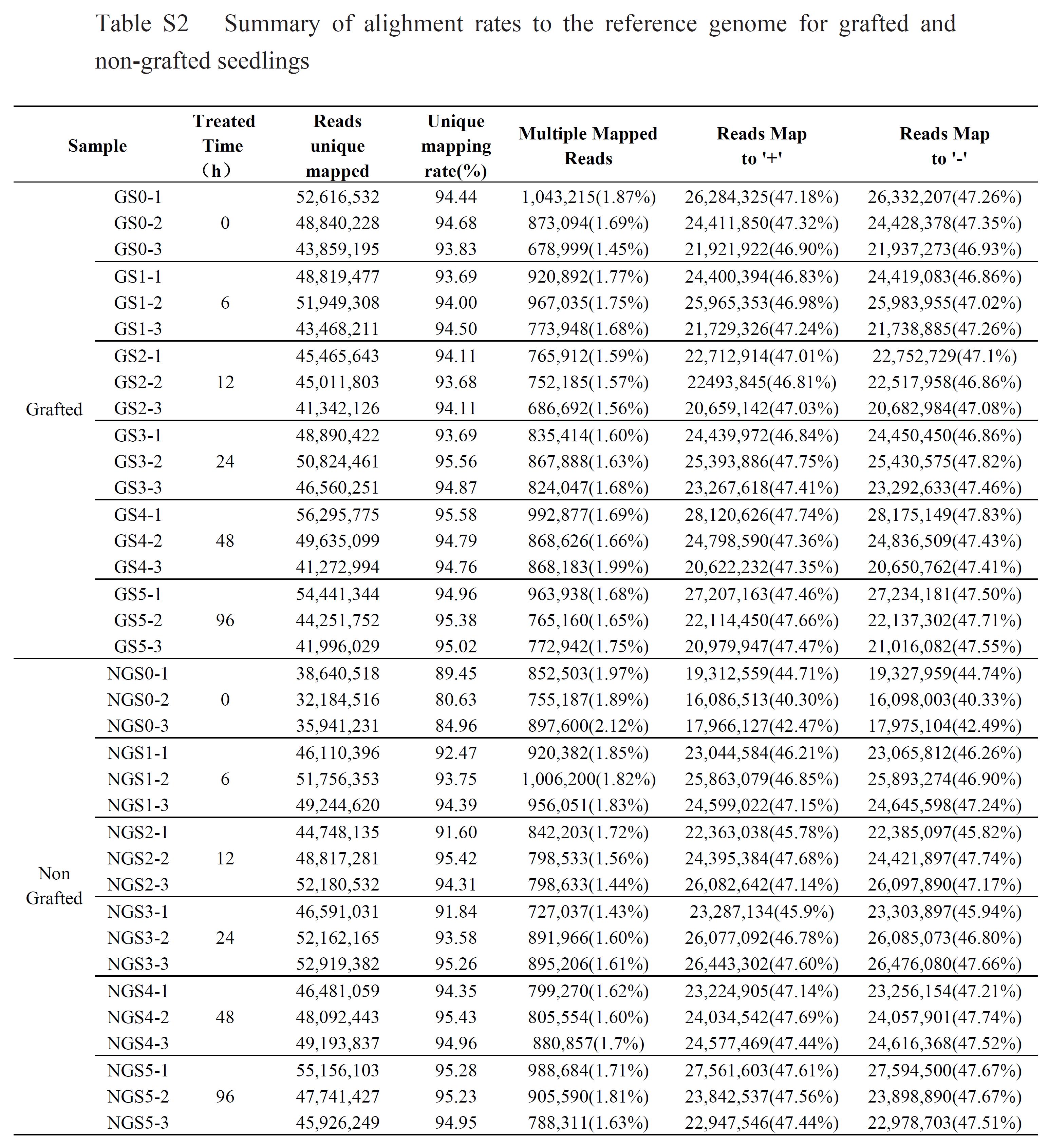

Supplement: Supplementary file 1 [file DataSheet_1.zip › additional file/Table S2.jpg]

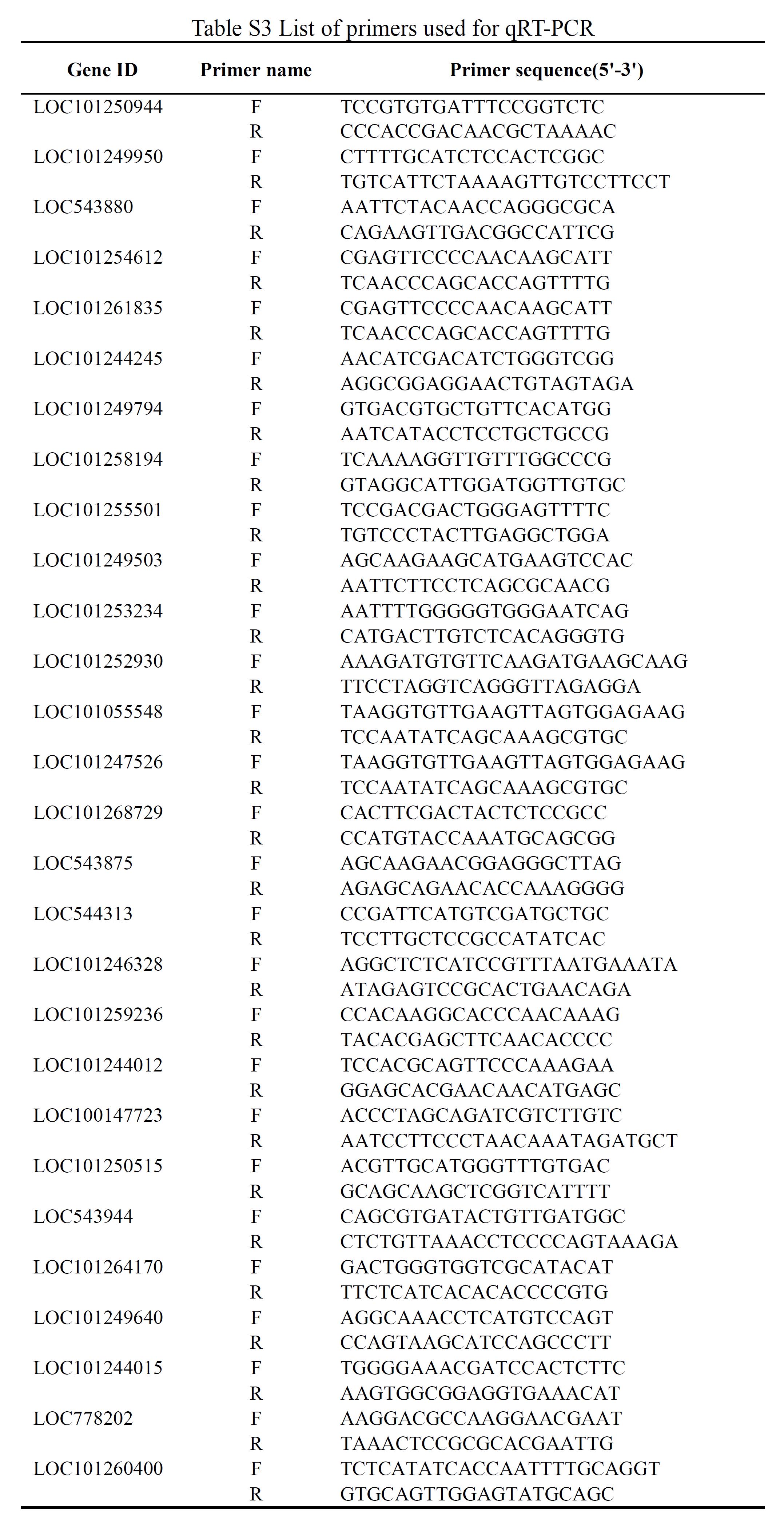

Supplement: Supplementary file 1 [file DataSheet_1.zip › additional file/Table S3.jpg]
